# *Holothuria polii* Extract as a Potential Anticoccidial Agent: Evidence of Its MUC2 Regulatory Impact in Murine Jejunum

**DOI:** 10.3390/vetsci11100490

**Published:** 2024-10-10

**Authors:** Youssef A. El-Sayed, Ahmed E. Abdel-Moneim, Rania G. Taha, Mona F. Khalil, Rewaida Abdel-Gaber, Felwa A. Thagfan, Esam S. Al-Malki, Mohamed A. Dkhil

**Affiliations:** 1 Department of Zoology and Entomology, Faculty of Science, Helwan University, Cairo 11759, Egypt; yousef.ahmed@science.helwan.edu.eg (Y.A.E.-S.); ahmed_abdelmoneim@science.helwan.edu.eg (A.E.A.-M.); mfkhalil@science.helwan.edu.eg (M.F.K.); mohameddkhil@science.helwan.edu.eg (M.A.D.); 2 Biological and Geological Sciences Department, Faculty of Education, Ain Shams University, Roxy, Heliopolis, Cairo 11757, Egypt; 3 Department of Zoology, College of Science, King Saud University, Riyadh 12372, Saudi Arabia; 4 Department of Biology, College of Science, Princess Nourah Bint Abdulrahman University, Riyadh 11564, Saudi Arabia; fathagfan@pnu.edu.sa; 5 Department of Biology, College of Science in Zulfi, Majmaah 11952, Saudi Arabia; e.almalki@mu.edu.sa

**Keywords:** eimeriosis, *Holothuria polii*, GC-MS, oxidative stress, goblet cells, MUC2 gene

## Abstract

**Simple Summary:**

Natural products offer significant potential in the fight against parasitic diseases. Coccidiosis is a disease caused by the *Eimeria* genus of parasites that infect many animals all over the world. The development of resistance to anticoccidial medications has refocused attention on finding new compounds for alternative therapeutic approaches. The purpose of this study was to determine the efficacy of *Holothuria polii* extract (HpE) in treating eimeriosis in mice. The results indicated that animals treated with 200 mg/kg of HpE showed a significant decrease in oocyst count and higher expression of the MUC2 gene. In conclusion, HpE functions as an anticoccidial substance.

**Abstract:**

*Eimeria* is a parasite that causes coccidiosis in a variety of animals, leading to nutritional imbalances, issues with food digestion and absorption, low weight, and intestinal inflammation of varying degrees in its hosts. Anticoccidial medication resistance has become a significant obstacle to disease control efforts, leading to a renewed focus on discovering novel chemicals for alternative therapeutic approaches. The purpose of this study was to determine the efficacy of *Holothuria polii* extract (HpE) in treating eimeriosis in mice. Five groups of mice were studied, with *E. papillata* sporulated oocysts (1 × 10^3^) used to infect three groups experimentally. The first group was treated with 200 mg/kg of HpE, the second group with Amprolium (120 mg/kg), and the third group was left untreated as a control. The fourth and fifth groups were uninfected, with one of them treated with 200 mg/kg of HpE and the other used as a negative control. The results revealed that HpE has 10 phytochemical compounds according to GC-MS analysis. Treatment with HpE resulted in a substantial decrease in oocyst output in feces. Also, the parasitic stages showed morphometric alterations, with reductions in the measurements compared with the infected, untreated mice. Moreover, expression of the mucin gene (MUC2) was higher in treated mice compared to infected ones, with significant increases in goblet cell numbers, which provided additional evidence for the activity of HpE as an anticoccidial product. In conclusion, there are a wide variety of natural products and many have been employed in folk medicine for treating a range of parasitic diseases.

## 1. Introduction

Coccidiosis is recognized to be one of the main disorders that restrict the growth and feed conversion efficiency of infected animals [1]. The *Eimeria* genus is described as a worldwide parasite, as it is widespread among vertebrate hosts, with over 1700 species reported [2]. It causes coccidiosis in a variety of animals of agricultural importance, especially affecting the poultry and cattle industries, as the parasite causes a reduction in weight and poor feed efficiencies [3]. Additionally, eimeriosis can cause several health problems, including growth retardation, appetite loss, anemia, anorexia, diarrhea, immunodeficiency, and in extreme cases, death [4,5]. By animals encountering infected excrement, the parasite transfers from one animal to another easily. This direct fecal–oral life cycle allows the parasite to proliferate quickly among vulnerable hosts [2]. When a sporulated oocyst (the infectious stage of the *Eimeria* species) is ingested by a new host, it hatches to produce eight sporozoites [6]. These sporozoites attack animal intestinal mucosa cells, rapidly dividing to generate merozoites within a meront. This causes extensive local and systemic inflammation and tissue damage [7].

Natural products have been essential in the development of drugs to combat pathogens, especially for infectious disorders and cancer [8,9]. To eliminate or control the spread of parasites, many researchers have tried to find alternative agents against eimeriosis, such as natural products, instead of using chemical drugs which have adverse effects on animal health [10,11]. Most of these agents are derived from natural sources, like plants, and are then chemically altered to provide them with more resistance to certain infections that affect humans [12].

Sea cucumbers are a large and diverse group of worm-like, soft-bodied echinoderms. They are found throughout the world’s oceans, with approximately 1500 species of 25 different families [13]. They have economic and health benefits and are known to have medicinal properties [14,15] because they are rich in minerals, particularly calcium, magnesium, iron, and zinc, and important nutrients such as vitamins A, B1 (thiamine), B2 (riboflavin), and B3 (niacin) [16].

In the present study, the activity of sea cucumber “*Holothuria polii*” methanolic extract has been determined to be an anticoccidial agent against infection by *Eimeria* in mice. During infection, the parasite induces the oxidation process, causing the production of large amounts of free radicals which put the animal under oxidative stress that leads to damage to the infected cells [17,18]. Some previous studies have used biological agents as antioxidants against murine eimeriosis. Alkhudhayri et al. demonstrated that selenium nanoparticles (SeNps) have antioxidant effects against eimeriosis [10]. Also, Abdel-Tawab et al. showed that the plant leaf extract “*Moringa oleifera*” could reduce oxidative stress levels and damage caused by *Eimeria* in mice [19]. To our knowledge, the current study is the first one to determine the efficacy of HpE as an anticoccidial agent and as a regulatory agent for the MUC2 gene in goblet cells in murine jejunum that were infected with *Eimeria papillata*.

## 2. Materials and Methods

### 2.1. Extraction of Holothuria polii

Sea cucumbers (*H. polii*) were collected from the Mediterranean Sea coast in Alexandria, Egypt. The samples’ taxonomic identity was confirmed with reference to Purcell et al. [20]. After washing sea cucumbers under running tap water, their body walls were cut into small pieces and their internal organs were removed. The body walls of *H. polii* were dried then powdered using an electric blender. According to the procedure outlined by Dakrory et al. [21], this powder was extracted by maceration with 70% methanol (MeOH). This mixture was continuously stirred then incubated at 4 °C for 24 h. After that, filtration was conducted after centrifugation for 15 min at 5000 rpm. The supernatant was concentrated at 50 °C under decreased pressure using a rotatory evaporator. The collected *H. polii* extract (HpE) powder was freeze-dried and stored at −80 °C in order to prepare two doses of HpE, 400 and 200 mg/kg.

### 2.2. Gas Chromatography-Mass Spectrometry (GC-MS) of HpE

The phytochemical analysis of HpE was executed out in accordance with the Kanthal et al. [22] suggested protocol. A Thermo Scientific 7000D Triple Quadrupole GC-MS instrument was used for the analysis. The chromatogram obtained was analyzed with MS, and peaks were identified by comparing collected mass spectra for substances to those in the GC-MS data system’s Wiley/NBS mass spectral database.

### 2.3. Experimental Design

Twenty-five male Swiss Albino mice of the *Mus musculus* species (10 ± 2 weeks old, weighing 22 ± 2 g) were obtained from VACSERA, Helwan, Cairo, Egypt, reared in pathogen-free conditions and fed a commercial pellet diet with tap water. The animals were housed in plastic cages with regulated temperatures (23 ± 5 °C), humidity, and a 12/12 light-dark cycle. They were given free access to commercial pellet food and tap water for one week prior to the trial beginning. Animals were divided into five separate groups of five mice each, as follows: The first group served as a negative control, comprised of uninfected and untreated mice. The second group consisted of non-infected, treated mice who received HpE at a dose of 200 mg/kg body weight. The third group was the positive control, which consisted of infected and untreated mice. The fourth group included infected mice treated with HpE at a dose of 200 mg/kg of body weight. Finally, the fifth group included infected and treated mice with Amprolium at a dose of 120 mg/kg body weight [23]. As reported by Abdel-Tawab et al., all groups, except the first and second, received an oral inoculation of 1 × 10^3^ *E. papillata* sporulated oocysts [19]. A preliminary dose_response study was conducted to identify the most effective dosage of HpE, as shown in Supplementary Figure S1.

### 2.4. Number of Oocysts 

On the fifth day post-infection (p.i.), when oocyst shedding was at its peak, animals from the third, fourth, and fifth groups were separated to collect fresh fecal pellets from each mouse. In a modified McMaster slide chambers, oocysts were counted [3]. In brief, feces were collected and weighted for each mouse. The feces were then suspended in a saturated saline solution (0.9% NaCl) in a weight/volume ratio of 1:15, and thoroughly mixed until completely mixed. Filtration through a sieve and two layers of gauze removed the bulk materials. The oocysts in 20 squares across both chambers were counted, and this total was multiplied by 100 to estimate the number of expelled *E. papillata* oocysts per gram of feces. The average number of oocysts shed was calculated for both treated and untreated groups.

### 2.5. Oocyst Dimentional Analysis 

*E. papillata* oocysts (both sporulated and non-sporulated) were imaged with an Olympus compound microscope and a CP72 digital camera (Olympus Corporation, Tokyo, Japan). Duszynski et al. devised the technique used to define the main features of morphology [24]. The calibrated ocular micrometer was used to collect data from 50 oocysts and 50 sporocysts.

### 2.6. Number of Parasitic Stages

The jejunum was separated, rinsed with phosphate- buffered saline (pH = 7.4), and quickly fixed in a 10% solution of neutral buffered formalin at 23 ± 2 °C for 24 h. The tissues were dehydrated with ethanol in an ascending sequence. The tissue samples were cleared in xylene, fixed in paraffin wax, and sectioned at 5 μm thickness on glass slides using a rotary microtome (Reichert-Jung, 820 H; Leica Biosystem, Buffalo Grove, IL, USA). The tissue sections were subsequently deparaffinized using xylene as well as stained with hematoxylin and eosin (H&E) [25]. The slides were mounted and dried before examination [26]. They were examined and imaged with an Olympus compound microscope and a CP72 digital camera (Olympus Corporation, Tokyo, Japan). The histopathological changes were observed and prepared for better illustrations. ImageJ 1.53e software (Wayne Rasband and contributors, National Institute of Health, Bethesda, MD, USA) was used to measure the photographed H&E stained section.

### 2.7. The Response of Goblet Cells during Eimeriosis

After deparaffinization and processing, tissue sections with a thickness of 5 µm were stained with Alcian blue for 30 min. The sections were washed several times with distilled water before being dehydrated with an ascending series of ethanol. Finally, the sections were mounted using DPX. The goblet cells’ number in each jejunum was determined by counting at least ten well-oriented villus crypt units (VCUs). The results were reported as the mean number of goblet cells per villus [27].

### 2.8. Oxidative Status in Jejunum

The jejuna of mice from the control, infected, and infected-treated groups were prepared to evaluate changes in oxidative state. They were immediately weighted and homogenized in an ice-cold solution containing 50 mM Tris-HCl with 300 mM sucrose. The final product was centrifuged at 500× *g* at 4 °C for 10 min, yielding a 10% (wt/v) jejunal homogenate [28]. The supernatant produced from the centrifuged samples was used for further biochemical investigation.

The NO assay was carried out using an ELISA kit from Kamiya Biomedical Company (catalog number MBS723386), which employs a competitive enzyme immunoassay approach. Samples were initially incubated with the enzyme conjugate and NO standards provided by the manufacturer, as per the methodology. The competitive binding process was allowed to occur, then the wells were cleansed to remove any unbound compounds. A substrate solution was then added to start the color development, which was halted after the required incubation time. The optical density was measured at 450 nm with a microplate reader.

### 2.9. Gene Expression Analysis

The QIAamp RNeasy Mini kit (Qiagen, Hilden, Germany, GmbH) was used to extract RNA from jejunal tissue. Tubes were placed in adapter sets that were fastened to the clamps of the Qiagen tissue lyser to homogenize the materials. The disruption was performed in a two-minute high-speed shaking step (30 Hz). The cleared lysate was treated with one volume of 70% ethanol, and the procedures were carried out in accordance with the QIAamp RNeasy Mini kit methodology for purifying total RNA from animal tissues. To remove any remaining DNA, the digestion was conducted on a column with DNase.

The primers (Metabion, Planegg, Germany) were used in a 25 µL reaction with 0.25 µL of RevertAid Reverse Transcriptase (200 U/µL) (Thermofisher), 12.5 µL of the 2x QuantiTect SYBR green PCR Master Mix (Qiagen, Germany, GmbH), 0.5 µL of each primer (Table S1) at a concentration of 20 pmol, 8.25 µL of water, and 3 µL of RNA template. The reaction was conducted using an Agilent Stratagene MX3005P QPCR system (California, USA). The Stratagene MX3005P program was used to generate amplification curves and CT data. To determine gene expression variance in the RNA, the “ΔΔCt” method was employed, as described by Yuan et al. [29]. This approach involved comparing the CT values of each sample to those of the positive control group, with β-actin used as the housekeeping gene for normalization.

### 2.10. Statistical Analysis

Group differences were evaluated using one-way analysis of variance (ANOVA). The Duncan post hoc test was applied to compare the means of the infected, infected treated, and control groups. Data analysis was performed with SigmaPlot^®^ version 11.0 (Systat Software, Inc., Chicago, IL, USA).

## 3. Results

### 3.1. Chemical Analysis of HpE

**Figure 1:**
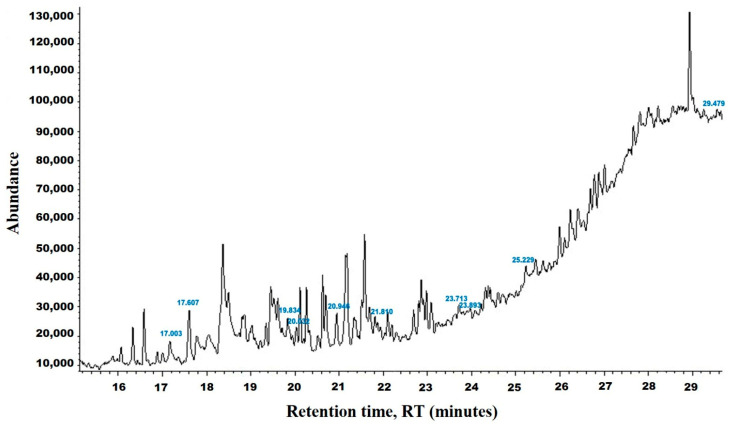
The GC-MS chromatogram of methanolic HpE.

The chemical analysis was performed by gas chromatography_mass spectrometry (GC-MS) (Figure 1, Table 1) and revealed that methanolic HpE included 10 chemical substances with varying molecular weights (MW) at varied peak regions and retention times (Table 1). The given peaks were recorded for 2,4-Di-tert-butylphenol, Dodecanoic acid, Tetradecanoic acid, Pentadecanoic acid, Palmitoleic acid, n-Hexadecanoic acid, 9,12-Octadecadienoic acid (Z,Z)-, Octadecanoic acid, 5,8,11,14- Eicosatetraenoic acid, methyl ester, (all-Z)-, and Squalene.

### 3.2. Oocyst Output

**Figure 2:**
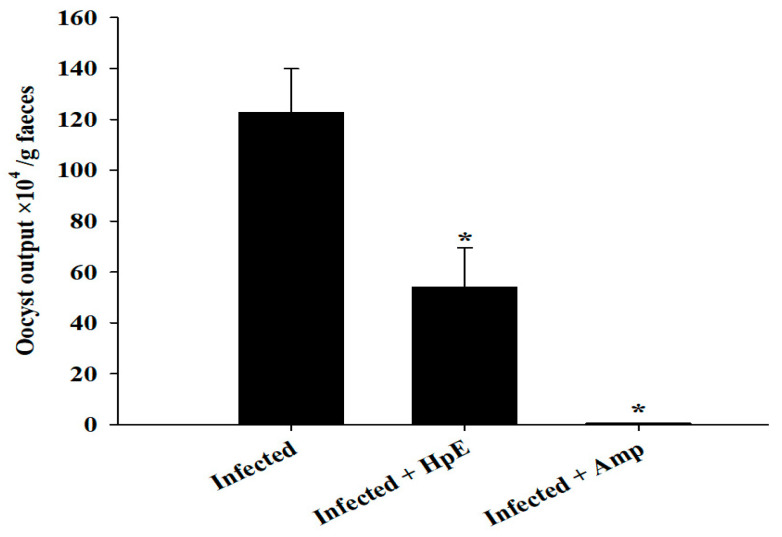
Oocyst output in mice infected with *E. papillata* and in the infected groups treated with 200 mg/kg HpE or 120 mg/kg Amprolium on day 5 p.i. Values are presented as the mean ± SD. * indicates significance (*p* ≤ 0.05) compared to the infected group.

Coccidial infection in mice with *E. papillata* resulted in oocyst discharge in fecal pellets, with a maximal level of 122.63 × 10^4^ ± 17.22 × 10^4^ oocysts per gram of feces on day 5 p.i. in the infected group. The oocyst expulsion after treatment with 200 mg/kg HpE was 53.97 × 10^4^ ± 15.45 × 10^4^ oocysts per gram of feces, and 0.43 × 10^4^ ± 0.13 × 10^4^ oocysts per gram of feces for the Amprolium reference drug with significant differences (*p* ≤ 0.01) from the control (Figure 2).

### 3.3. Oocyst Morphometry

**Figure 3:**
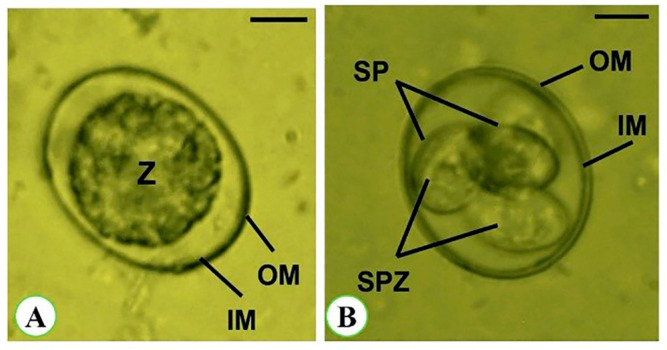
*Eimeria papillata* oocysts. (**A**) Non-sporulated oocyst. (**B**) Sporulated oocyst. Scale bar = 5 µm. OM: outer membrane, IM: inner membrane of the oocyst, Z: zygote, SP: sporocysts, SPZ: sporozoites.

Oocysts are sub-spherical in shape, with a thick bi-layered wall around them. The unsporulated oocyst measures 17.62 (16.41–20.65) µm long and 15.52 (12.99–16.57) µm broad. It is enclosed by a double-layered wall with an inner zygote measuring 14.44 (13.00–15.67) µm long and 13.42 (11.24–15.29) µm wide. The zygote length/width (L/W) index is 1.08. The sporulated oocyst is 19.63 (18.24–22.67) µm long and 17.22 (14.57–19.55) µm wide, with an L/W index of 1.14. The oocysts are tetrasporocystic and disporozoic. The sporocysts are ellipsoidal in shape and have a single-layered wall. The sporocysts are 9.22 (7.68–11.57) µm long and 6.11 (5.68–7.51) µm wide, having an L/W index of 1.51. The sporocyst residuum consists of tiny granules that are distributed among sporozoites. These sporozoites contain sausage-shaped nuclei and a refractile body (Figure 3 and Table 2).

### 3.4. Parasitic Stages

**Figure 4:**
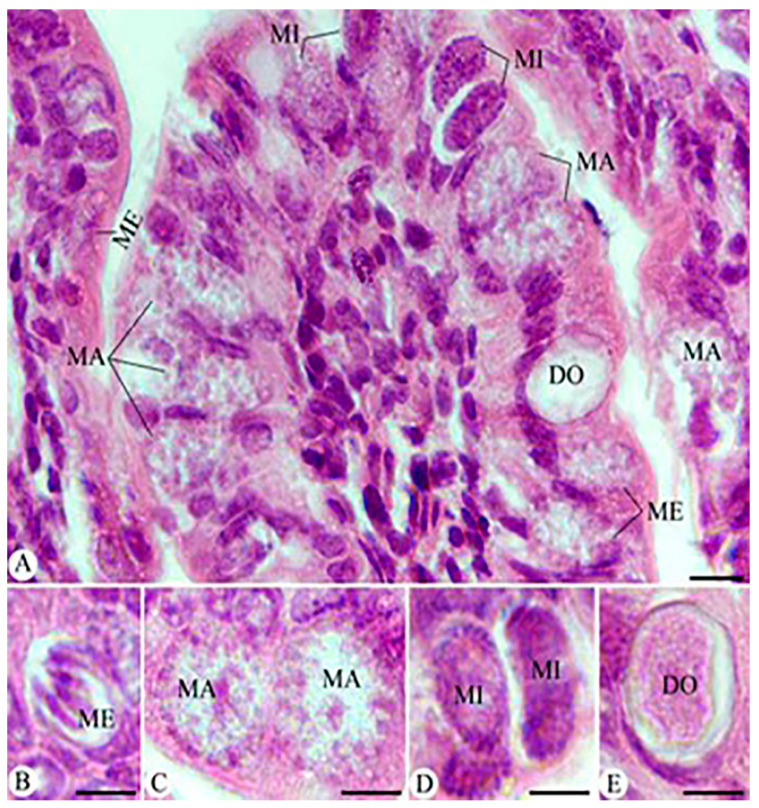
Hematoxylin and eosin (H&E)-stained sections of the jejunum infected with *E. papillata* on day 5 post-infection, illustrating various developmental stages (**A**–**E**). ME: meronts, MA: macrogamont, MI: microgamont, DO: developing oocyst. Scale bar = 10 µm.

Mice infected with *E. papillata* oocysts developed several parasite stages such as meronts, microgamonts, macrogamonts, and developing oocysts in jejunum epithelial cells, as shown in H&E stained sections (Figure 4). Morphometric alterations in the developmental changes of *E. papillata* in infected and treated groups are reported in (Figure 4, and Table 2), as the measures for microgamonts, macrogamonts, and developing oocysts are substantially decreased in the infected group after HpE treatment, as well as in the Amprolium-treated group.

### 3.5. The Response of Goblet Cells (GCs) during Eimeriosis

**Figure 5:**
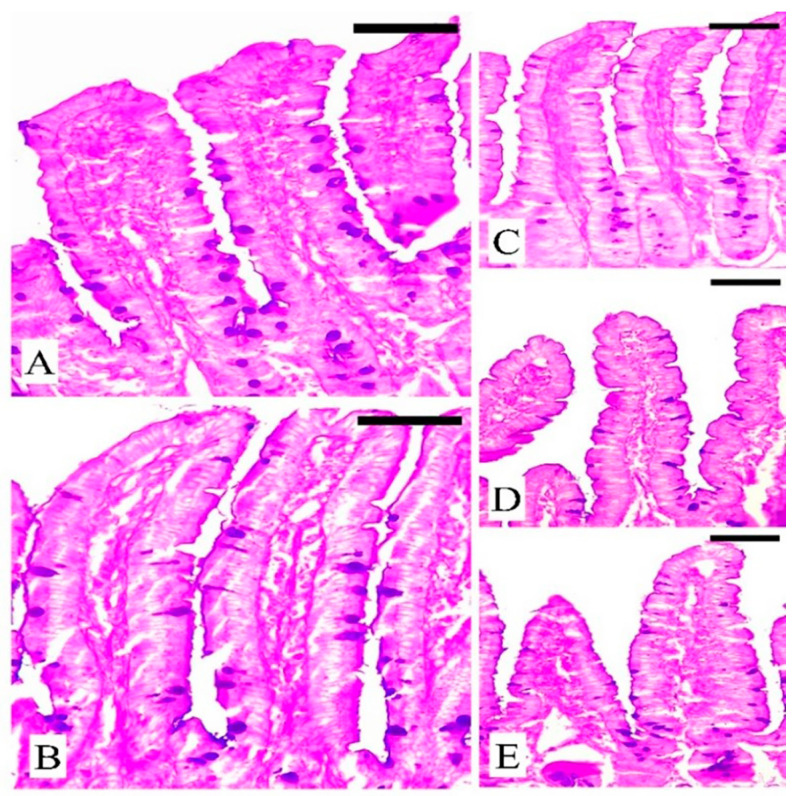
Sections of mouse jejunum on day 5 post-infection, highlighting goblet cells. (**A**) Non-infected jejunum from the control group. (**B**) Non-infected jejunum treated with HpE only. (**C**) Infected jejunum with *E. papillata*. (**D**) Jejunum of infected mice treated with HpE. (**E**) Jejunum of infected mice treated with Amprolium. Sections were stained with Alcian blue. Scale bar = 10 µm.

**Figure 6:**
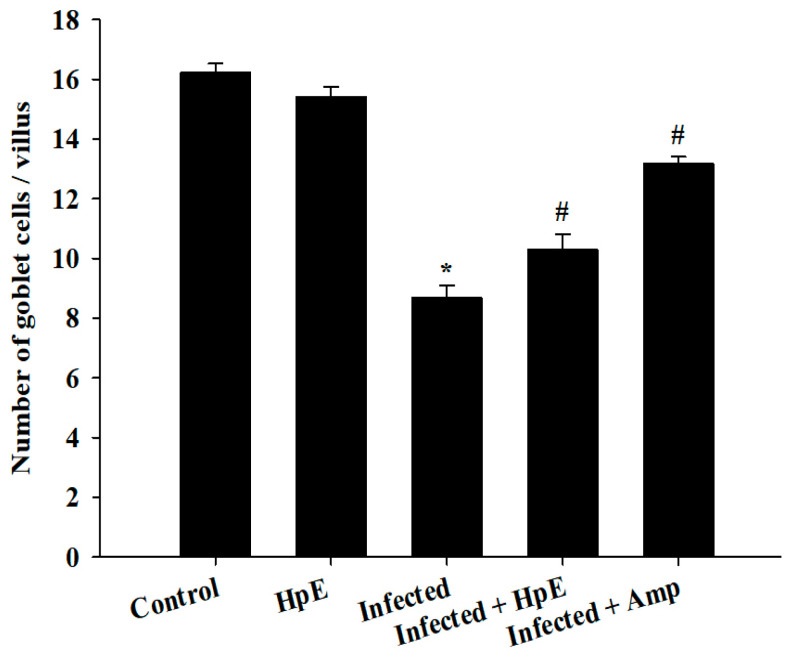
Changes in the number of jejunal GCs in the control group, non-infected-HpE group, infected group, infected -treated group with 200 mg/kg HpE, and infected -treated group with 120 mg/kg Amprolium. Values are the means ± SD. * significance (*p* ≤ 0.05) against the control group, # significance (*p* ≤ 0.05) against the infected group.

Microscopic investigation of Alcian blue-stained jejunal sections (Figure 5) showed that mice infected with *E. papillata* had considerably fewer GCs (8.68 ± 0.41/VCU) than the control group (16.22 ± 0.31/VCU) (*p* ≤ 0.05), and the non-infected group that treated with HpE only showed a significant increase (12.90 ± 0.30/VCU) in GCs. The number of GCs in mice treated with Amprolium was significantly (*p* ≤ 0.05) higher (13.19 ± 0.22/VCU) than in mice treated with HpE (10.28 ± 0.22/VCU) (Figure 5 and Figure 6).

### 3.6. Assessment of NO Oxidative Stress Biomarkers

**Figure 7:**
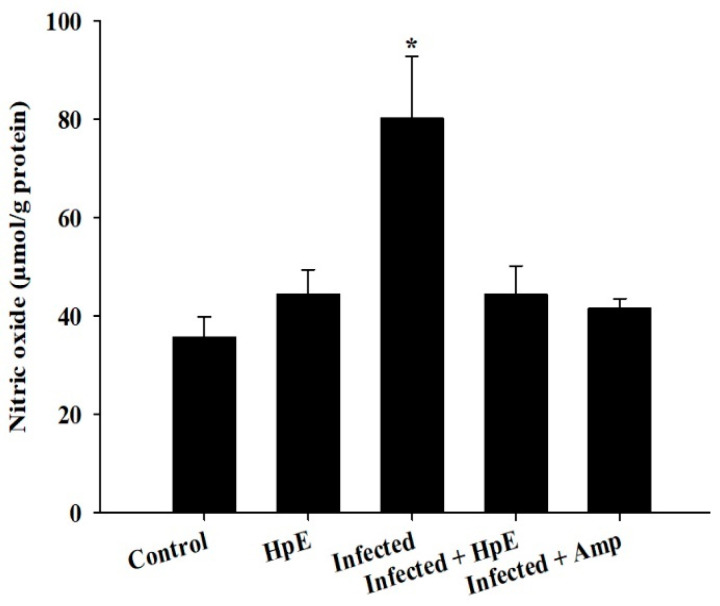
Changes in the level of jejunal NO. Values are the means ± SD. * significance (*p* ≤ 0.05) against the control group.

*E. papillata* infection significantly increases NO levels; however, HpE treatment reduces NO levels in infected mice. Infected mice had more significant (*p* ≤ 0.05) NO levels (80.33 ± 12.52 µmol/g protein) compared to the control group (35.77 ± 4.14 µmol/g protein) and the HpE group (44.49 ± 4.94 µmol/g protein). However, infected mice treated with HpE had lower NO levels (47.61 ± 5.65 µmol/g protein) and those treated with Amprolium had lower levels (41.54 ± 2.02 µmol/g) (*p* ≤ 0.01) (Figure 7).

### 3.7. Mucin Genes Analysis

**Figure 8:**
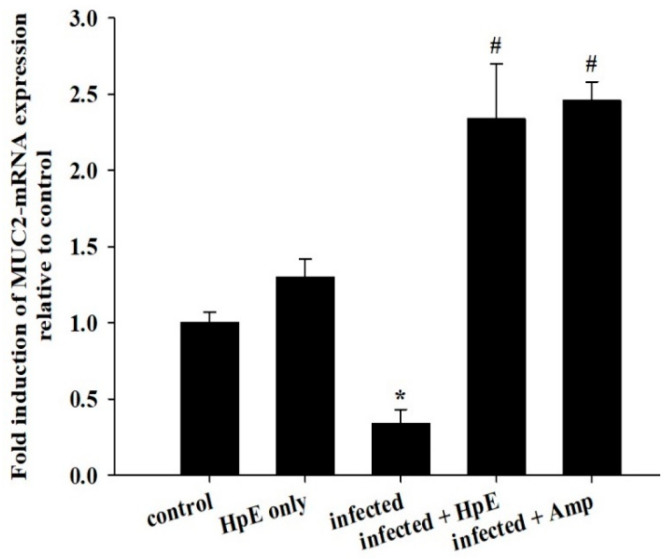
Effect of HpE on the mRNA expression of MUC2 in jejunal samples from *E. papillata*-infected mice. The expression values, obtained by RT-PCR analysis, were normalized to the β-actin mRNA level and are presented as fold induction (log2 scale) relative to the mRNA level in the control. * indicates significance compared to the control group, and # indicates significance compared to the infected group.

On day 5 p.i., *E. papillata* infection reduced the mucin gene MUC2 expression in the jejunum in infected mice. HpE treatment significantly upregulated (*p* ≤ 0.01) the expression of the MUC2 gene from 1.30 ± 0.12 to 2.34 ± 0.36 fold (Figure 8). The results were adjusted to the β-actin mRNA level and shown as fold induction (in log2 scale) related to the mRNA level in the control by RT-PCR.

## 4. Discussion

Coccidiosis is one of the world’s most serious livestock diseases, caused by parasitic protozoa of the *Eimeria* genus [30]. It affects agriculturally important animals, particularly in the poultry and cattle industries, where the parasite leads to weight loss and reduced feed efficiency [3]. Anticoccidial drugs are commonly used to treat coccidiosis, although overuse can lead to resistance [31]. Natural products, such as HpE, offer a promising alternative due to their diverse bioactive compounds that can target multiple pathways, reducing the likelihood of resistance development. Furthermore, they are generally perceived as safer and more sustainable options, which is particularly important for the livestock industry where coccidiosis poses a significant economic burden [7,8]. The current results revealed that HpE contains ten important and vital compounds that have a great effect as antioxidants, anti-inflammatory and potential therapeutic effects. One of these compounds is 2,4-Di-tert-butylphenol which has a role as a bacterial metabolite, an antioxidant and a marine metabolite [32]. Dodecanoic acid has antimicrobial properties [33]. Moreover, Pentadecanoic acid has broad activities relevant to protecting cardiometabolic, immune, and liver health [34], and Palmitoleic acid is able to regulate different metabolic processes such as β-cell proliferation [35]. Also, according to [36], n-Hexadecanoic acid may help in designing specific inhibitors of phospholipase A (2) as anti-inflammatory agents. Squalene is a compound that exhibits as anti-inflammatory and has antioxidant properties involved in regulation of lipid metabolism [37]. The use of HpE could not only help to mitigate the spread of drug-resistant strains but also provide a cost-effective and environmentally friendly solution for managing coccidiosis in livestock [14]. This is crucial as over-reliance on synthetic anticoccidials has led to resistance and residues in animal products, raising both health and economic concerns. Therefore, the integration of natural products into coccidiosis management strategies could enhance the sustainability and resilience of livestock production systems. Eimerian parasites have a preference for parasitizing particular intestinal segments based on their genus [38]. The jejunum of the mouse intestine is the preferred site for *E. papillata* growth, which can result in severe enteritis and elevated oxidative stress [39]. The present results revealed a considerable shift in the infected host’s oxidative status, as the parasite increased the level of nitric oxide (NO). Under normal conditions, the creation and elimination of reactive free radicals are in dynamic equilibrium. When the number of free radicals created surpasses the antioxidant defense system’s ability to defend, this balance can be disrupted [17]. According to Esch et al., *Eimeria* infection causes a disturbance in the antioxidant defense system, which contributes to the harmful effects on cells [40]. Some authors assessed the antioxidant status of animals infected by *Eimeria* species. For example, Koinarski et al. [17] investigated the blood antioxidant levels of broiler chickens infected with *Eimeria acervuline*. They observed variations in the investigated enzymes and non-enzymatic parameters to show that broiler chickens infected with *E. acervulina* experienced oxidative stress after infection and had a reduced antioxidant state. Additionally, Al-Otaibi et al. [9] recorded a decrease in NO in infected mice by *Eimeria*.

Infectivity distinguishes two types of oocysts; the first type is the infective sporulated oocyst which can live for up to 50 months in the environment (including feces, litter, feed, and even soil) prior to invading the host. The second type is the unsporulated oocyst (the non-infective form which can stay in the caecum of a host for 7 months) [30,41]. Under suitable circumstances (such as oxygen, moisture, and temperature), unsporulated oocysts can become sporulated oocysts; this process is referred to as sporulation [31]. Our investigation indicated a significant reduction in the amount of oocyst production in treated animals with 200 mg/kg of HpE as compared to the control ones, which was accompanied by a rise in the number of goblet cells. The less potent effect of HpE than Amprolium on oocyst output and goblet cell counts could be due to differences in their mechanisms of action. Amprolium directly interferes with the parasite’s thiamine metabolism, effectively reducing oocyst output, while HpE likely exerts its effects through immunomodulatory and antioxidant properties, which might not directly impact oocyst production to the same extent. The above-mentioned results accord with those reported by Abdel-Gaber et al. [11] and Dkhil et al. [18] as they reported that infected mice treated with Nanoselenium exhibited a decrease in the oocyst count excreted in feces by 80%. The same results were obtained by Abdel-Tawab et al. who treated coccidiosis with *Moringa oleifera* leaf extract [19]. In the present work, qRT-PCR analysis revealed that *Eimeria* infection resulted in a down-regulation of MUC2 gene expression, whereas the HpE-treated animals exhibited overexpression of the same gene which resulted in more goblet cells in the jejunum. Goblet cells are widely known for secreting mucus, which acts as a barrier against a number of infections, such as *Eimeria* [38]. Goblet cells have also been shown to modify the components of mucus in response to pathogenic stimuli, in addition to multiplying and expanding in size [42]. Regarding the overexpression of MUC2, although it is known to protect the intestinal barrier, its overexpression alone may not be sufficient to further decrease nitric oxide levels. This could be because the reduction in nitric oxide is more closely associated with the modulation of pro-inflammatory cytokines and other oxidative stress pathways, which may not be directly influenced by MUC2 expression levels. Further studies exploring the interplay between MUC2 expression and immune response modulation could provide deeper insights into this complex relationship. Linh et al. [38] observed modifications and reductions in the goblet cells’ number following *Eimeria vermiformis* infection in mice. They also observed infiltrations of lymphoid cells as lymphocytes and plasma cells into the lamina propria in addition to villous atrophy.

The present results agree with Dkhil et al. [18] who discovered the anti-eimerial effect of nanomagnesium through the regulation of the gene expression of MUC2 and MUC4 in infected mice. Alkhudhayri et al. [10] found that Nanoselenium (SeNPs) can control the gene expression of MUC2, IL-1β, IL-6, IFN-γ, and TNF-α in the jejunum of mice infected with *E. papillata*. Thus, in the current experiment, the observed decrease in goblet cells may actually have an influence on the ability of host to defend itself through a different method. Ultimately, our results show that a sea cucumber methanolic extract of dose 200 mg/kg possesses anticoccidial efficacy in comparison with the commercial anticoccidial Amprolium. Further research should focus on larger sample sizes and an in-depth dose-response study to optimize HpE dosing and better understand its mechanism of action.

## 5. Conclusions

*Holothuria polii* extract may be a promising anticoccidial agent for the treatment of coccidiosis. Over decades, natural ingredients have become a vital resource for the development of novel medications. However, further investigations are needed to study the impact of infection by coccidiosis on the gene expression that is related to defense mechanisms in animals and to estimate the role of new natural products as anti-eimeriosis agents.

## Figures and Tables

**Table 1:** Identification of chemical compounds by GC-MS in HpE.

t_R (min)_	Compound	MW	Peak Area	Peak Area (%)	Structure	Formula
17.049	2,4-Di-tert-butylphenol	206	126,864	5.90	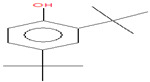	C_14_H_22_O
17.638	Dodecanoic acid	200	736,245	34.23	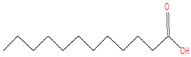	C_12_H_24_O_2_
19.932	Tetradecanoic acid	228	224,314	10.43	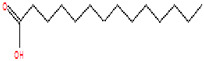	C_14_H_28_O_2_
20.005	n-Hexadecanoic acid	256	182,407	8.48	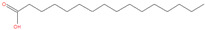	C_16_H_32_O_2_
20.994	Pentadecanoic acid	242	380,350	17.68	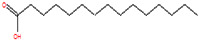	C_15_H_30_O_2_
21.815	Palmitoleic acid	254	91,151	4.24	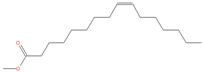	C_16_H_30_O_2_
23.667	9,12-Octadecadienoic acid (Z,Z)-	280	116,629	5.42	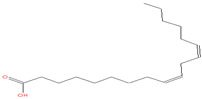	C_18_H_32_O_2_
23.898	Octadecanoic acid	284	69,120	3.21	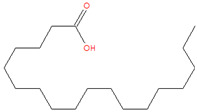	C_18_H_36_O_2_
25.193	5,8,11,14-Eicosatetraenoic acid, methyl ester, (all-Z)-	318	187,140	8.70	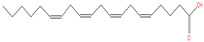	C_21_H_34_O_2_
29.443	Squalene	410	36,859	1.71	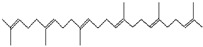	C_30_H_50_

**Table 2:** Morphometery of *E. papillata* oocysts.

Oocysts	Sporulated Oocyst	Non-Sporulated Oocyst
Length (µm)	17.62 ± 2.18	19.63 ± 2.27
Width (µm)	15.52 ± 1.84	17.22 ± 2.49
L/W index	1.08	1.14

## Data Availability

The raw data supporting the conclusions of this article will be made available by the authors on request.

## Supplementary Materials

The following supporting information can be downloaded at: https://www.mdpi.com/article/10.3390/vetsci11100490/s1, Figure S1: Oocyst output in mice infected with E. papillata and in the infected groups treated with 200 & 400 mg/kg HpE and 150 mg/kg Amprolium on day 5 post-infection. Values are mean ± SD. * indicates significance (*p* < 0.05) compared to the infected group.; Table S1: Primer sequences used for real-time quantitative reverse transcriptase polymerase chain reaction (qRT-PCR).
