# Supplementary material for: Holothuria polii Extract as a Potential Anticoccidial Agent: Evidence of Its MUC2 Regulatory Impact in Murine Jejunum
Source: Vet Sci. 2024 Oct 10;11(10):490. doi: 10.3390/vetsci11100490 (PMC11512319; doi:10.3390/vetsci11100490)
Supplement: Supplementary file 1 [file vetsci-11-00490-s001.zip › vetsci-3236183-supplementary.pdf]

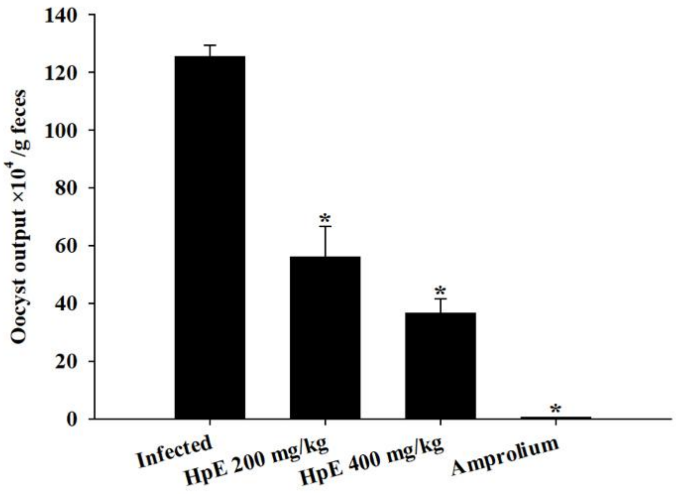

Figure 1S Oocyst output in mice infected with *E. papillata* and in the infected groups treated with 200 & 400 mg/kg HpE and 150 mg/kg Amprolium on day 5 post-infection. Values are mean ± SD. * indicates significance ($P < 0.05$) compared to the infected group.

Table. S1 Primer sequences used for real-time quantitative reverse transcriptase polymerase chain reaction (qRT-PCR).

Target	Primer sequence (5'→ 3')
**Mucin (MUC2)**	F-TTCGGCACGAGCAACTTTG<br>R-GGCAGGACACCTTGTCATTG
***β*-actin**	F-GCT ACA GCT TCA CCA CCA CA<br>R-AAG GAA GGC TGG AAA AGA GC
